# Supplementary material for: Improving Skin-to-Skin Practice for babies in Kangaroo Mother Care in Malawi through the use of a customized baby wrap: A randomized control trial
Source: PLoS One. 2020 Mar 19;15(3):e0229720. doi: 10.1371/journal.pone.0229720 (PMC7082027; doi:10.1371/journal.pone.0229720)
Supplement: S1 Appendix — (DOCX) [file pone.0229720.s001.docx]

### S1 Appendix: Sample size calculation

The original primary outcome for the study was *average* *rate of weight gain*. The study sample size was calculated to accommodate rate of weight gain. We hypothesized that mothers using the customized wrapper would practice skin-to-skin contact for greater duration and that the longer duration of skin-to-skin contact would increase the rate of weight gain. Specifically, we anticipated that the average rate of weight gain, measured in grams (g) per day while in hospital, would be 20% higher among babies of mothers using the customized wrap than those using the traditional wrap. This 20% difference was considered to be a clinically meaningful increase in the average weight gain, and it was also feasible to detect within the available resources for the study. Facility staff recruited for the study measured the weight of all enrolled babies upon admission to KMC and at the time of facility discharge using a digital infant scale with 20g accuracy. Birthweight data were extracted from the maternity registers. Data collectors visited each mother-baby dyad discharged from the facility at their home within 7-15 days of discharge, and they weighed the baby using the same type of scale. Average rate of weight gain was measured in g/day for two periods: (1) from the time of KMC admission to the time of facility discharge, and (2) from time of facility discharge to the 7- to 15-day follow-up visit.

A specific limitation of calculating appropriate weight gain as a percentage of gain/loss from birthweight for our study was that the scales used to measure weight had an accuracy of ±20g and the technique varied by healthcare providers. Data recorded on birthweight from maternity registers appeared to be rounded. Aggregating weight gains and losses across our study population was difficult to interpret, given the discrepancy of the data. Prior systematic reviews looking at the effect of KMC on a multitude of health outcomes have found weak linkages between weight gain and KMC [3].
