# Supplementary material for: Improving Skin-to-Skin Practice for babies in Kangaroo Mother Care in Malawi through the use of a customized baby wrap: A randomized control trial
Source: PLoS One. 2020 Mar 19;15(3):e0229720. doi: 10.1371/journal.pone.0229720 (PMC7082027; doi:10.1371/journal.pone.0229720)
Supplement: S2 Appendix — (DOCX) [file pone.0229720.s002.docx]

### S2 Appendix: Data collection tools

**FORM 0: ELIGIBILITY ASSESSMENT – MALAWI KMC WRAPPER STUDY**

0

**STUDY ARM (CIRCLE ONE): 1. INTERVENTION 2. CONTROL IDENTIFYING INFORMATION**

| **1.** | Facility ID | 1. QECH 2. THYOLO DH 3. MACHINGA DH | | | |
| --- | --- | --- | --- | --- | --- |
| **2.** | Child Assessment ID (*for data editor to assign*) |  | | | |
| **3.** | Name of child*(do not enter in database)* |  | | | |
| **4.** | Mother’s name*(do not enter in database)* |  | | | |
| **5.** | Village where child’s family is from (and give approximate distance to HF in km) | Village name | | | |
| **6.** | Estimated distance from child’s village to hospital | **1** | ≤10 kms | **2** | > 10 kms |
| **7.** | Date of Assessment *(dd/mm/yyyy)* | */ /* | | | |
| **8.** | Child’s date of birth *(dd/mm/yyyy)* | */ /* | | | |
| **9.** | Child’s date of admission to KMC | / / | | | |
| **10.** | Child’s age in days at admission to KMC | **1** | <7 days | **2** | ≥7 days |
| **11.** | Child’s sex | **1** | Male | **2** | Female |

**CLINICIAN’S ASSESSMENT**

| **12.** | Child’s birthweight in g | | g | | | |
| --- | --- | --- | --- | --- | --- | --- |
| **13.** | Child has a neurological problem like neural tube defects and hydrocephalus or other major congenital abnormalities | | **1** | Yes | **2** | No |
| **14.** | Child has major complications in need special care (e.g. severe respiratory problems, severe infections) | | **1** | Yes | **2** | No |
| **15.** | Child weighed <800g at birth (check #12) | | **1** | Yes | **2** | No |
| **16.** | Child’s mother died during labour. | | **1** | Yes | **2** | No |
| **17.** | Child ≥7 days old at admission to KMC (check #8 above) | | **1** | Yes | **2** | No |
| **EXCLUSION CRITERIA** | | | | | | |
| **18.** | Child’s family resides >10 km from hospital (check #6) | | **1** | Yes | **2** | No |
| **19.** | Does child have any complications? (yes to any of #13-17) | | **1** | Yes | **2** | No |
| **INCLUSION CRITERIA** | | | | | | |
| **20.** | Does the child meet inclusion criteria? *(birthweight <2000g and admitted to facility-based KMC )* | | **1** | Yes | **2** | No |
| **ELIGIBILITY FOR ENROLMENT** | | | | | | |
| **21.** | Is this infant eligible for enrolment into the trial? | | **1** | Yes | **2** | No |
| **22.** | Consent provided to participate in the study? | | **1** | Yes (go to Form 1) | **2** | No (go to 23) |
| **23.** | If parent/caretaker not willing to sign consent, provide standard of care, and document the reason for not consenting: | | | | | |
|  | **a** | Parent/caretaker too busy | **1** | Yes | **2** | No |
|  | **b** | Does not like research | **1** | Yes | **2** | No |
|  | **c** | Other (specify) | **1** | Yes | **2** | No |

**I have checked the form for missing variable and inconsistencies:**

| **24.** | Clinician’s Initials |  | Date *(dd/mm/yyyy)* | / / |
| --- | --- | --- | --- | --- |
| **25.** | Form checked by research coordinator |  | Date *(dd/mm/yyyy)* | / / |
| **26.** | Data Entry 1 (Editor) |  | Date *(dd/mm/yyyy)* | */ /* |
| **27.** | Data Entry 2 |  | Date *(dd/mm/yyyy)* | */ /* |

FORM ID: **🗌🗌🗌** MOTHER ID: **🗌🗌🗌🗌🗌** BABY ID: **🗌🗌🗌🗌🗌** Twin ID: **🗌🗌🗌🗌🗌**

*Note: in case of multiples, complete one form for each child*

| **#** | **Item** | **Response** |
| --- | --- | --- |
| **1.** | Hospital | QECH 1  THYOLO DH 2  MACHINGA DH 3 |
| **2.** | Study arm | INTERVENTION 1  CONTROL 2 |
| **3.** | Date of enrollment (dd/mm/yyyy) | **🗌🗌**/**🗌🗌**/**20🗌🗌** |
| **4.** | Name of data collector |  |
| **Maternal information (from maternity register)** | | |
| **5.** | Date of admission to hospital (dd/mm/yyyy) | **🗌🗌/🗌🗌/20🗌🗌** |
| **6.** | Parity | Parity………**🗌🗌** |
| **7.** | Place of delivery | This hospital 1  Other health facility 2  Outside facility (describe below) 3 |
| **8.** | Any maternal complications at delivery (*circle all that apply)* | No complications 0  Antenatal postpartum hemorrhage 1  Postpartum hemorrhage 2  Obst/prolonged labour 3  (Pre) Eclampsia 4  Sepsis 5  Ruptured uterus 6  Other(specify) 7 |
| **9.** | Mother’s HIV status | HIV negative 1  HIV positive 2  Unknown 3 |
| **Baby information (from maternity register)** | | |
| **10.** | Date of delivery (dd/mm/yyyy) | **🗌🗌**/**🗌🗌**/**20🗌🗌** |
| **11.** | Baby gender | Male 1  Female 2 |
| **12.** | Mode of delivery | Vaginal 1  Cesarean 2  Vacuum Extraction 3 |
| **13.** | Gestational age in completed weeks | Completed weeks………**🗌🗌** |

| **#** | **Item** | **Response** |
| --- | --- | --- |
| **14.** | Multiple births | Singleton 1  Twin 2  Triplet 3  Other (specify: ) 4 |
| **15.** | Birth weight (in grams) | **🗌🗌🗌🗌 g** |
| **16.** | Complications at birth (*circle all that apply*) | No complications 0  Birthweight <2500g 1  Prematurity 2  Asphyxia 3  Sepsis 4  Other(specify) 5 |
| **KMC initiation information (from KMC register/records)** | | |
| **17.** | Date of initiation of KMC (dd/mm/yyyy) | **🗌🗌**/**🗌🗌**/**20🗌🗌** |
| **18.** | Number of days between date of delivery and date of initiation to KMC (*compare date of delivery with date of KMC initiation; record 0 if admitted on same*  *day as delivery*) | #of days **🗌🗌** |
| **19.** | Weight at initiation to KMC | **🗌🗌🗌🗌 g** |

**I have checked the form for missing variable and inconsistencies:**

| **20.** | Form checked by research coordinator |  | Date *(dd/mm/yyyy)* | / / |
| --- | --- | --- | --- | --- |
| **21.** | Data Entry 1 (Editor) |  | Date *(dd/mm/yyyy)* | / / |
| **22.** | Data Entry 2 |  | Date *(dd/mm/yyyy)* | / / |

| FORM ID: **🗌🗌🗌** MOTHER ID: **🗌🗌🗌🗌🗌** BABY ID: **🗌🗌🗌🗌🗌** Twin ID: **🗌🗌🗌🗌🗌**  **COHORT (CIRCLE ONE): 1. INTERVENTION 2. CONTROL** | | | | | | | | | | | | |
| --- | --- | --- | --- | --- | --- | --- | --- | --- | --- | --- | --- | --- |
| **Background information** | | **Day** | | **Day** | | **Day** | | | **Day** | | **Day** | |
| **1.** | Date of observation (dd/mm/yy) | / / | | / / | | / / | | | / / | | / / | |
| **2.** | Name of data collector |  |  |  |  |  |  | |  |  |  |  |
| **3.** | Child’s name |  | |  | |  | | |  | |  | |
| **4.** | Child’s gestational age in completed weeks |  | |  | |  | | |  | |  | |
| **5.** | Child’s chronological age in days |  | |  | |  | | |  | |  | |
| **Anthropometry** | |  | |  | |  | | |  | |  | |
| **6.** | Child’s weight in g at time | I I I I I | | I I I I I | | I I I I I | | | I I I I I | | I I I I I | |
| **KMC position/bonding** | |  | |  | |  | | |  | |  | |
| **7.** | KMC Positioning (not positioned=1, not well-positioned =2, well-positioned =3) |  | |  | |  | | |  | |  | |
| **8.** | Use of KMC wrapper (using Laerdal wrapper = 1, using traditional wrap = 2, no wrap/not in KMC = 3) (If 1 or 2 skip to 11 otherwise go to 10 |  | |  | |  | | |  | |  | |
| **9.** | If not using wrap, cause: uncomfortable = 1, prefers traditional wrap = 2, prefers Laerdal = 3 not practicing KMC = 4, other = 4 (specify) |  | |  | |  | | |  | |  | |
| **Time in skin-to-skin position** | | DAY TIME | NIGHT TIME | DAY TIME | NIGHT TIME | DAY TIME | NIGHT TIME | | DAY TIME | NIGHT TIME | DAY TIME | NIGHT TIME |
| **10.** | Time period observed (give starting time and end time) | Start: End: | Start: End: | Start: End: | Start: End: | Start: End: | | Start: End: | Start: End: | Start: End: | Start: End: | Start: End: |
| **11.** | Number of hours observed during day time (6am-6pm) | I I I  hours |  | I I I  hours |  | I I I  hours | |  | I I I  hours |  | I I I  hours |  |
| **12.** | Duration (in hours) child kept in skin-to-skin position in day- time (6am-6pm) | I I I  hours |  | I I I  hours |  | I I I  hours | |  | I I I  hours |  | I I I  hours |  |
| **13.** | Number of hours observed in evening/night time (>6pm to  <6am) |  | I I I  hours |  | I I I  hours |  | | I I I  hours |  | I I I  hours |  | I I I  hours |
| **14.** | Duration (in hours) child kept in skin-to-skin position in night- time (>6pm to <6am) |  | I I I  hours |  | I I I  hours |  | | I I I  hours |  | I I I  hours |  | I I I  hours |
| **15.** | Total number of hours observed in 24 hour period (11+13) | I I I hours I I I hours | | I I I hours I I I hours | | I I I hours I I I hours | | | I I I hours I I I hours | | I I I hours I I I hours | |
| **16.** | Total duration of skin-to-skin positioning during 24 hour period (12+14) | I I I hours I I I hours | | I I I hours I I I hours | | I I I hours I I I hours | | | I I I hours I I I hours | | I I I hours I I I hours | |

| **17.** | Type [KMC stopped=1, Intermittent = 2, Continuous=3] (If 1 or 3 skip to 19) |  | |  | |  | |  | |  | |
| --- | --- | --- | --- | --- | --- | --- | --- | --- | --- | --- | --- |
| **18.** | If intermittent, cause: mother sick =1, provider not available = 2, others = 3 |  | |  | |  | |  | |  | |
| **19.** | Any other family members providing KMC? (Yes =1 (indicate who; no = 2) |  | |  | |  | |  | |  | |
| **Feeding practices (**Both observation and mother’s responses**)** | |  | |  | |  | |  | |  | |
| **20.** | Feeding Instruction: Breast feeding=1, Nothing per orally = 2, Artificial milk=3 |  | |  | |  | |  | |  | |
| **21.** | I/V fluid: Y=1, N=2 |  | |  | |  | |  | |  | |
| **22.** | NG Tube feeding Y=1, N=2 |  | |  | |  | |  | |  | |
| **23.** | Cup feeding Y=1, N=2 |  | |  | |  | |  | |  | |
| **24.** | Breast feeding Y=1, N=2 ( if not breast feed skip to 31 ) |  | |  | |  | |  | |  | |
| **25.** | Frequency of BF/24 h |  |  |  |  |  |  |  |  |  |  |
| **26.** | Exclusive BF=1, Partly BF=2,Not BF=3 |  | |  | |  | |  | |  | |
| **27.** | If not EBF, what food given |  | |  | |  | |  | |  | |
| **Attachment and positioning while breast feeding** | |  | |  | |  | |  | |  | |
| **28.** | **Attachment evaluation:** Good = 1 (chin touching breast, more areola above than below mouth, mouth wide open); Not well = 2, No attachment = 3) |  | |  | |  | |  | |  | |
| **29.** | **Positioning evaluation:** Good = 1 (straight head & body, close to mother, whole body full supported, facing breast with nose opposite to nipple), Not good = 2, Not BF = 3) |  | |  | |  | |  | |  | |
| **Drugs and other treatments** | |  | |  | |  | |  | |  | |
| **30.** | Injection antibiotics (Y=1 – give type; N=2) |  | |  | |  | |  | |  | |
| **31.** | Injection vitamin K (Y=1, N=2) |  | |  | |  | |  | |  | |
| **32.** | Other - specify |  | |  | |  | |  | |  | |
| **Status update/remarks:** | |  | |  | |  | |  | |  | |
| **33.** | Status: Continue KMC in KMC unit =1 Referred to Neonatal Unit =2, Returned to KMC Unit=3, Absconded=4, DOR=5, Death=6, Discharged from facility KMC=7 |  | |  | |  | |  | |  | |
| **Comments: use space to provide any further observations** | |  | |  | |  | |  | |  | |

**I have checked the form for missing variable and inconsistencies:**

| **24.** | Research officer’s Initials |  | Date *(dd/mm/yyyy)* | / / |
| --- | --- | --- | --- | --- |
| **25.** | Form checked by research coordinator |  | Date *(dd/mm/yyyy)* | / / |
| **26.** | Data Entry 1 (Editor) |  | Date *(dd/mm/yyyy)* | / / |
| **27.** | Data Entry 2 |  | Date *(dd/mm/yyyy)* | / / |

FORM ID: **🗌🗌🗌** MOTHER ID: **🗌🗌🗌🗌🗌** BABY ID: **🗌🗌🗌🗌🗌** Twin ID: **🗌🗌🗌🗌🗌**

Hello, My name is and I am with the KMC study at hospital. As you know, we are

conducting a study on behalf of recently delivered mothers with small/preterm babies to explore adherence to KMC practice. You indicated your willingness to participate in the study and I am here to ask your opinions about KMC practices. May I take your few minutes to ask few questions related to this? Thank you.

***Circle outcome:*** *1=Mother agrees; 2=Mother refuses (give reason: )*

*Fill out 1 form per mother (even if she has twins or triplets)*

| **#** | **Item** | **Response** |
| --- | --- | --- |
| **1.** | Hospital | QECH 1  THYOLO DH 2  MACHINGA DH 3 |
| **2.** | Study arm | INTERVENTION 1  CONTROL 2 |
| **3.** | Date of data collection | **🗌🗌**/**🗌🗌**/**20🗌🗌** |
| **4.** | Name of data collector |  |
| **Maternal background information** | | |
| **5.** | What is your age (in years)? | Years **🗌🗌** |
| **6.** | What is your highest level of education? | No formal schooling 1  Some primary school 2  Completed primary school 3  Some secondary school 4  Completed secondary school 5  Formal school post‐secondary 6 |
| **7.** | What is your marital status? | Married 1 |
|  |  | Single 2 |
|  |  | Divorced/separated 3 |
|  |  | Widowed 4 |
| **8.** | What is your employment? | None 0  Skilled/Self‐employed 1  Salaried work 2  Agriculture/farmer 3  Business… 4  Other… 5 |
| **9.** | What is your monthly family income (*convert* | <10 .......................................................... 1 |
|  | *estimate to US dollars*)? | 10‐15 .......................................................2 |
|  |  | 15‐30 .......................................................3 |
|  |  | 30‐60 .......................................................4 |
|  |  | >60 .......................................................... 5 |

| **#** | **Item** | **Response** |
| --- | --- | --- |
| **Maternal health status during pregnancy** | | |
| **10.** | Did you take malaria prophylaxis while you were pregnancy with NAME? | Yes 1  No… 2 |
| **11.** | Did you experience fever/malaria while you were pregnant with NAME? | Yes 1  No… 2>>#13 |
| **12.** | Did you receive treatment for malaria while you were pregnant with NAME? | Yes 1  No… 2 |
| **Mother’s feedback/perceptions on material used for KMC wrap (to be administered on Day 3 after mother has**  **initiated KMC with traditional or customized wrap)** | | |
| **13.** | Record what the mother is using to support the baby in KMC position | KMC study wrapper (Laerdal) 1  Traditional wrap (chitenje) 2  Other (specify: ) 3 |
| **14.** | Are you comfortable using the wrap to keep your baby in skin‐to‐skin position? | Yes 1  No (explain why not below) 2 |
| **15.** | How long did it take you to feel comfortable using the wrap? | Was comfortable on Day 1 1  Was comfortable on Day 2 2  Was comfortable on Day 3 3  Still not comfortable using wrap 4 |
| **16.** | Are you able to tie the wrap by yourself or do you need someone else to help you? | Can tie by herself 1  Needs someone else to help 2 |
| **17.** | Is the wrap comfortable when you are breastfeeding? If no, why not? | Yes 1  No (explain why not below) 2 |
| **18.** | Is the wrap comfortable when you are sleeping? If no, why not? | Yes 1  No (explain why not below) 2 |
| **19.** | Do you feel that the wrap keeps the baby in a secure position? If no, why not? | Yes 1  No (explain why not below) 2 |

| **#** | **Item** | **Response** |
| --- | --- | --- |
| **20.** | Is it easy to monitor your baby’s condition while using the wrap? If no, why not? | Yes 1  No (explain why not below) 2 |
| **21.** | What do you like about the wrap you are using to keep your baby in KMC position? |  |
| **22.** | What do you dislike/wish were different about the wrap you are using to keep your baby in KMC? |  |
| **23.** | Do you think the KMC wrap you are using is acceptable for fathers or male family members to use? Why or why not? | Yes (explain why below)… 1  No (explain why not below) 2 |
| **24.** | Have you faced any difficulties using the KMC wrap you are using? If yes, please explain. | Yes (explain why below)… 1  No… 2 |
| **Question for mothers in intervention arm (LGH wrapper) only** | | |
| **25.** | Do you prefer to use the KMC wrapper over the chitenje to keep the baby in STS position? Why or why not? | Yes (explain why below)… 1  No (explain why not below) 2  Don’t know/unsure… 3 |
| *Thank mother for her time and participation. Remind her that someone from the study will be visiting her at her home*  *within 7‐10 days to follow‐up with her and the baby.* | | |

**I have checked the form for missing variable and inconsistencies:**

| **1.** | Form checked by research coordinator |  | Date *(dd/mm/yyyy)* | / / |
| --- | --- | --- | --- | --- |
| **2.** | Data Entry 1 (Editor) |  | Date *(dd/mm/yyyy)* | / / |
| **3.** | Data Entry 2 |  | Date *(dd/mm/yyyy)* | / / |

FORM ID: **🗌🗌🗌** MOTHER ID: **🗌🗌🗌🗌🗌** BABY ID: **🗌🗌🗌🗌🗌** Twin ID: **🗌🗌🗌🗌🗌**

Hello, My name is and I am with the KMC study at hospital. As you know, we are

conducting a study on behalf of recently delivered mothers with small/preterm babies to explore adherence to KMC practice. You indicated your willingness to participate in the study and I am here to discuss about KMC practice before you go home. May I take your few minutes to ask few questions related to this? Thank you.

***Circle outcome:*** *1=Mother agrees; 2=Mother refuses (give reason: )*

*Note: in case of multiples, complete one form for each child (repeat questions 4‐22 for each additional child to have info on vital status and STS; ensure additional pages are securely stapled together)*

| **#** | **Item** | **Response** |
| --- | --- | --- |
| **1.** | Hospital | QECH 1  THYOLO DH 2  MACHINGA DH 3 |
| **2.** | Study arm | INTERVENTION 1  CONTROL 2 |
| **3.** | Date of data collection | **🗌🗌**/**🗌🗌**/**20🗌🗌** |
| **4.** | Name of data collector |  |
| **Discharge information (from KMC facility records)** | | |
| **5.** | Date of discharge from KMC (dd/mm/yyyy) | **🗌🗌/🗌🗌/20🗌🗌** |
| **6.** | Total number of days in KMC (from KMC admission to KMC discharge) | # of days… **🗌🗌** |
| **7.** | Status at discharge from KMC | Met discharge criteria 1  Died 2  Referred to higher centre 3  Left against medical advice 4  Other 5 |
| **8.** | Child’s gestational age at time of discharge from KMC | Completed weeks………**🗌🗌** |
| **9.** | Child’s age in days at time of discharge from KMC | Age in days… **🗌🗌** |
| **10.** | Child’s weight at admission/initiation on KMC (g) | **🗌🗌🗌🗌 g** |
| **11.** | Child’s weight at time of discharge (g) | **🗌🗌🗌🗌 g** |
| **12.** | Total weight change between admission to KMC and discharge from KMC (#10‐#9) | **🗌🗌🗌🗌 g** |

| **#** | **Item** | **Response** |
| --- | --- | --- |
| **13.** | Is the child able to breast‐feed? | Yes 1  No 2 |
| **14.** | Is the child receiving feeds through cup and spoon? | Yes 1  No 2 |
| **15.** | Breastfeeding status at the time of discharge | Exclusive breastfeeding 1  Predominantly breastfeeding 2  Partial breastfeeding 3  No breastfeeding 4 |
| **16.** | While in KMC, did the baby become sick and need treatment? | Yes 1  No 2 >>#18 |
| **17.** | What treatment did he or she receive? | Injection (antibiotics) 1  Oxygen 2  CPAP placement (bubble) 3  IV fluids 4  Bag and mask ventilation 5  Phototherapy 6  Other 7  Don’t know 8 |
| **18.** | Date of first follow‐up visit? (record date when mother is expected to return to the health facility) | **🗌🗌**/**🗌🗌**/**20🗌🗌** |
| **Observation of mother and baby at time of discharge** | | |
| **19.** | Is baby in skin‐to‐skin position at the time of discharge? | Yes 1  No 2 |
| **20.** | Is baby wearing a cap/hat? | Yes 1  No 2 |
| **21.** | Is baby wearing socks/booties? | Yes 1  No 2 |
| **22.** | What is mother using for a KMC wrap? | KMC study wrapper (Laerdal) 1  Traditional wrap (chitenje) 2  Other (specify: ) 3 |
| **KMC practices** | | |
| **23.** | What challenges did you face practicing skin‐to‐skin while in the facility? |  |
| **24.** | When did you find it easier to do skin‐to‐skin, was it during the day‐time or at night? | Day‐time 1  Night‐time 2  Both are the same 3  Don’t know 8 |

| **#** | **Item** | **Response** |
| --- | --- | --- |
| **25.** | During the day‐time, on average, how much time was your baby in skin‐to‐skin position?  *(Read the response options to the mother)* | Not at all 1  Some of the day (less than half) 2  More than half the day 3  Nearly all the day 4 |
| **26.** | Can you tell me how many hours you usually placed the baby in KMC position each day (on the days you practiced KMC)?  *(Record mother’s response and use the information to classify – if needed, read the response options to the mother)* | Classification:  4 hours or less… 1  5‐10 hours… 2  11‐19 hours… 3  20 or more hours… 4  Don’t know… 8 |
| **27.** | At what times did you take the baby off skin‐to‐skin position?  (*circle all mentioned; and prompt*) | To take a rest 1  To go to bathroom 2  For feeding 3  For sleeping 4  For visitors 5  When baby was crying/fussy 6  Other 7 |
| **28.** | How often did you practice skin‐to‐skin at night– every night, some nights, or not at all?  *(Read the response options to the mother)* | Not at all 1  Several nights 2  More than half the nights 3  Nearly every night. 4 |
| **29.** | During the night‐time, on average, how much time was your baby in skin‐to‐skin position?  *(Read the response options to the mother)* | Not at all 1  Some of the night (less than half) 2  More than half the night 3  Nearly all the night 4 |
| ***Now I would like to know about your skin‐to‐skin practices in the last 24 hours (last full day in KMC).*** | | |
| **30.** | During the previous day‐time, for how much time was the baby in skin‐to‐skin position?  *(Read the response options to the mother)* | Not at all 1  Some of the day (less than half) 2  More than half the day 3  Nearly all the day 4 |
| **31.** | During the previous night‐time, for how much time was the baby in skin‐to‐skin position?  *(Read the response options to the mother)* | Not at all 1  Some of the night (less than half) 2  More than half the night 3  Nearly all the night 4 |
| **Support for KMC** | | |
| **32.** | When you were being trained on KMC, was any other family member involved? | Yes 1  No 2>>#34 |

| **#** | **Item** | **Response** |
| --- | --- | --- |
| **33.** | Who else was involved?  (*Circle all that apply; prompt – ‘anyone else?’*) | Husband 1  Sister 2  Mother 3  Mother‐in‐law 4  Other (specify: ) 5 |
| **34.** | Did you have any family members providing help for you while you were in KMC? | Yes 1  No 2 >>#37 |
| **35.** | What help did they provide?  (*Circle all that apply; prompt ‘anything else?’)* | Held baby in skin‐to‐skin position 1  Provided food 2  Cared for children at home 3  Provided moral support 4  Other (specify: ) 5 |
| **36.** | Who helped you?  (*Circle all that apply; prompt – ‘anyone else?’*) | Husband 1  Sister 2  Mother 3  Mother‐in‐law 4  Other (specify: ) 5 |
| **37.** | What support did you receive for feeding from health facility staff while in KMC? |  |
| **38.** | What support did you receive for skin‐to‐skin positioning from health facility staff while in KMC? |  |
| **Intentions to practice KMC at home** | | |
| **39.** | Do you intend to continue practicing KMC when you return home? Why or why not? | Yes (explain why below) 1  No (explain why not below) 2 |
| **40.** | How prepared do you feel to practice KMC at home? | Very prepared 1  Somewhat prepared 2  Not very prepared 3  Don’t know 8 |

| **#** | **Item** | **Response** |
| --- | --- | --- |
| **Mother’s feedback/perceptions on material used for KMC wrap** | | |
| **41.** | Record what the mother is using to support the baby in KMC position | KMC study wrapper (Laerdal) 1  Traditional wrap (chitenje) 2  Other (specify: ) 3 |
| **42.** | Are you comfortable using the wrap to keep your baby in skin‐to‐skin position? | Yes 1  No (explain why not below) 2 |
| **43.** | Are you able to tie the wrap by yourself or do you need someone else to help you? | Can tie by herself 1  Needs someone else to help 2 |
| **44.** | Is the wrap comfortable when you are breastfeeding? If no, why not? | Yes 1  No (explain why not below) 2 |
| **45.** | Is the wrap comfortable when you are sleeping? If no, why not? | Yes 1  No (explain why not below) 2 |
| **46.** | Do you feel that the wrap keeps the baby in a secure position? If no, why not? | Yes 1  No (explain why not below) 2 |
| **47.** | Is it easy to monitor your baby’s condition while using the wrap? If no, why not? | Yes 1  No (explain why not below) 2 |
| **48.** | What do you like about the wrap you are using to keep your baby in KMC position? |  |
| **49.** | What do you dislike/wish were different about the wrap you are using to keep your baby in KMC? |  |
| **Satisfaction with KMC wrap received** | | |
| **50.** | Overall, how satisfied are you with the KMC wrap you received? Why or why not satisfied? | Very satisfied 1  Somewhat satisfied 2 |

| **#** | **Item** | **Response** |
| --- | --- | --- |
|  |  | Not very satisfied 3  Don’t know 8  Reason: |
| **51.** | Do you intend to continue using the KMC wrap you received when you go home? | Yes (explain why below) 1  No (explain why not below) 2 |
| **Question for mothers in intervention arm only (LGH wrapper)** | | |
| **52.** | Do you prefer the KMC wrapper over the chitenje to keep the baby in STS position? Why or why not? | Yes (explain why below) 1  No (explain why not below) 2  Don’t know/not sure… 3 |
| *Thank mother for her time and participation. Remind her that someone from the study will be visiting her at her home*  *within 7‐10 days to follow‐up with her and the baby.* | | |

**I have checked the form for missing variable and inconsistencies:**

|  | Form checked by research coordinator |  | Date *(dd/mm/yyyy)* | / / |
| --- | --- | --- | --- | --- |
|  | Data Entry 1 (Editor) |  | Date *(dd/mm/yyyy)* | / / |
|  | Data Entry 2 |  | Date *(dd/mm/yyyy)* | / / |

FORM ID: **🗌🗌🗌** MOTHER ID: **🗌🗌🗌🗌🗌** BABY ID: **🗌🗌🗌🗌🗌** Twin ID: **🗌🗌🗌🗌🗌**

Hello, My name is and I am with the KMC study at hospital. As you know, we are

conducting a study on behalf of recently delivered mothers with small/preterm babies to explore adherence to KMC practice. You indicated your willingness to participate in the study during your delivery at hospital. In

continuation of the study I am calling you to discuss about KMC practices at your home. May I take your few minutes to ask few questions related to this? Thank you.

***Circle outcome:*** *1= Mother agrees; 2=Mother refuses; 3=Unable to make contact*

*Note: in case of multiples, complete one form for each child (repeat first page for each additional child to have info on vital status and STS; ensure additional pages are securely stapled together)*

| **#** | **Item** | **Response** |
| --- | --- | --- |
| **1.** | Hospital | QECH 1  THYOLO DH 2  MACHINGA DH 3 |
| **2.** | Study arm | INTERVENTION 1  CONTROL 2 |
| **3.** | Date of discharge from KMC (dd/mm/yyyy)  *(Record from facility records)* | **🗌🗌/🗌🗌/20🗌🗌** |
| **4.** | Date of data collection | **🗌🗌**/**🗌🗌**/**20🗌🗌** |
| **5.** | Name of data collector |  |
| **Child’s vital status and weight at time of interview** | | |
| **6.** | Vital status | Alive 1 >>#8  Dead 2 |
| **7.** | When did this happen?  ***Record date of death and END INTERVIEW*** | **🗌🗌**/**🗌🗌**/**2015**  Don’t know 8  If cannot remember date, ask how many days after discharge: |
| **8.** | Child’s weight (g) at time of visit  *Weigh child and record weight in g* | **🗌🗌🗌🗌** g |
| **Observation of mother/family members and baby at time of interview** | | |
| **9.** | Is baby in skin‐to‐skin position? | Yes 1  No 2 |
| **10.** | Is baby wearing a cap/hat? | Yes 1  No 2 |
| **11.** | Is baby wearing socks/booties? | Yes 1  No 2 |
| **12.** | What is mother using for a KMC wrap? | KMC study wrapper (Laerdal) 1  Traditional wrap (chitenje) 2  Other (specify: ) 3 |

| **#** | **Item** | **Response** |
| --- | --- | --- |
| **Maternal interview** | | |
| *I would like to know more about your skin‐to‐skin practices since you were discharged from the hospital.* | | |
| **13.** | Are you still practicing skin‐to‐skin? | Yes… 1 >>#15  No… 2 |
| **14.** | Why are you no longer practicing skin‐to‐skin? (*Circle all that apply; prompt ‘anything else?’*) | Baby no longer needs KMC… 1  Too busy to practice KMC… 2  Uncomfortable… 3  People looking at you strangely… 4  Husband/other family not supportive…5 Health issues in mother/baby… 6  Other chores/responsibilities… 6  Negative attitude/remarks of people  in the community 7  Don’t know/not sure… 8  Other (specify 88 |
| **15.** | For how long did you practice skin‐to‐skin after you came home? | Never practiced KMC at home… 0  1‐2 days… 1  3‐4 days 2  5‐7+ days… 3  Don’t know… 8 |
| ***FOR RESPONDENTS NO LONGER PRACTICING KMC – SKIP TO #25*** | | |
| **16.** | How often do you practice skin‐to‐skin?  *(Read the response options to the mother)* | Every day… 1  5‐6 days per week… 2  3‐4 days per week… 3  1‐2 days per week… 4  Other (specify )… 88 |
| **17.** | Are you practicing skin‐to‐skin while doing household chores? | Yes 1  No (explain why not below) 2 |
| **18.** | Do you go out of your house with your baby in skin‐ to‐skin position? | Yes 1  No (explain why not below) 2 |

| **#** | **Item** | **Response** |
| --- | --- | --- |
| **19.** | What challenges have you faced practicing skin‐to‐ skin since you came home?  (*Circle all that apply; prompt ‘anything else?’*) | Other responsibilities/chores 1  Caring for other children 2  Cooking 3  People looking at you strangely 4  Husband/other family not supportive 5  Health issues of mother/baby 6  Negative attitude/remarks of people  in the community 7  Don’t know/not sure 8  Other (specify ) 9 |
| **20.** | When do you find it easier to do skin‐to‐skin ‐ during the day‐time or at night? | Day‐time 1  Night‐time 2  Both are the same 3  Don’t know 8 |
| **21.** | During the day‐time, on average, how much time is your baby in skin‐to‐skin position?  *(Read the response options to the mother)* | Not at all 1  Some of the day (less than half) 2  More than half the day 3  Nearly all the day 4 |
| **22.** | At what times do you take the baby off skin‐to‐skin position?  (*circle all mentioned; and prompt*) | To take a rest 1  To go to bathroom 2  For feeding 3  For sleeping 4  For visitors 5  When baby was crying/fussy 6  To go out of the house 7  To do household chores 8  Other ..... |
| **23.** | How often do you practice skin‐to‐skin at night– every night, some nights, or not at all?  *(Read the response options to the mother)* | Not at all 1  Several nights 2  More than half the nights 3  Nearly every night 4 |
| **24.** | During the night‐time, on average, how much time is your baby in skin‐to‐skin position?  *(Read the response options to the mother)* | Not at all 1  Some of the night (less than half) 2  More than half the night 3  Nearly all the night 4 |
| ***Now I would like to know about your skin‐to‐skin practices in the last 24 hours*** | | |
| **25.** | During the previous day‐time, for how much time was the baby in skin‐to‐skin position?  *(Read the response options to the mother)* | Not at all 1  Some of the day (less than half) 2  More than half the day 3  Nearly all the day 4 |

| **#** | **Item** | **Response** |
| --- | --- | --- |
| **26.** | During the previous night‐time, for how much time was the baby in skin‐to‐skin position?  *(Read the response options to the mother)* | Not at all 1  Some of the night (less than half) 2  More than half the night 3  Nearly all the night 4 |
| **Support for KMC at home and in the community** | | |
| **27.** | Have any family members provided help for you for KMC since you returned home? | Yes 1  No 2 >>#30 |
| **28.** | Who helped you?  (*Circle all that apply; prompt – ‘anyone else?’*) | Husband 1  Sister 2  Mother 3  Mother‐in‐law 4  Other (specify: ) 5 |
| **29.** | What help did they provide?  (*Circle all that apply; prompt ‘anything else?’)* | Held baby in skin‐to‐skin position 1  Provided food 2  Cared for children at home 3  Provided moral support 4  Household chores 5  Other (specify: ) 6 |
| **30.** | What has been the attitude of people in your family to KMC? |  |
| **31.** | Has anyone in the community (outside your family) provided support for you to practice KMC since you returned home? | Yes 1  No 2 >>#34 |
| **32.** | Who in the community helped you? | Friends 1  Women’s groups 2  Village leaders 3  Other (specify: ) 4 |
| **33.** | What help did they provide?  (*Circle all that apply; prompt ‘anything else?’)* | Held baby in skin‐to‐skin position 1  Provided food 2  Cared for children at home 3  Provided moral support 4  Household chores 5  Other (specify: ) 6 |
| **34.** | What has been the attitude of people in your community to KMC? |  |

| **#** | **Item** | **Response** |
| --- | --- | --- |
| **Feeding practices** | | |
| **35.** | Is your baby able to breastfeed? | Yes 1  No 2 |
| **36.** | Do you use cup and spoon to provide breast‐milk to your baby? | Yes 1  No 2 |
| **37.** | How often are you breast‐feeding your baby during the day‐time? | Hourly… 1  Every two hours… 2  Every three hours… 3  On demand… 4 |
| **38.** | How often are you breast‐feeding your baby during the night‐time? | Hourly… 1  Every two hours… 2  Every three hours… 3  On demand… 4 |
| **39.** | Since you were discharged from the facility, have you fed your baby anything other than breast‐milk? If yes, what else have you given the baby?  *(If yes, what else have you given the baby? Then list)* | Yes (describe below) 1  No 2 |
| **Mother and baby health issues** | | |
| **40.** | Have you experienced fever/malaria since you were discharged from the health facility? | Yes… 1  No… 2 |
| **41.** | Since you were discharged from KMC at the hospital, did your baby become sick? | Yes 1  No 2 |
| **42.** | Was the baby kept in skin‐to‐skin position when sick? If no, why not? | Yes 1  No (explain why not below) 2 |
| **43.** | Where did you seek care? | Hospital (QECH or Bwaila) 1  Other health facility 2  Name of other facility:  Traditional healer 3  Did not seek care 4 >>#46 |
| **44.** | Was baby ever admitted to the hospital or referred to the hospital whilst sick? | Yes 1  No 2 |
| **45.** | What treatment did he or she receive? Probe | Injection (antibiotics) 1  Oxygen 2  CPAP placement (bubble) 3  IV fluids 4  Bag and mask ventilation 5  Phototherapy 6  Other 7  Don’t know 8 |

| **#** | **Item** | **Response** |
| --- | --- | --- |
| **KMC follow‐up** | | |
| **46.** | Did you return to the hospital/health facility for follow‐up? | Yes… 1 >>#48  No… 2 |
| **47.** | What did the health worker do for the baby during the follow‐up visit?  (*Circle all that apply; prompt ‘anything else?’*) | Weighed the baby… 1  Asked about feeding 2  Asked about KMC practices 3  Checked baby’s temperature… 4  Gave advice about caring for baby… 5  Gave vaccination… 6  Other (specify )… 88  Don’t know/Not sure… 8 |
| **48.** | Did an HSA/community health worker visit you in your home to check on you and your baby? | Yes, HSA 1  Yes, other community worker 2  No 3>>#50 |
| **49.** | What did the community health worker do during the visit?  (*Circle all that apply; prompt ‘anything else?’*) | Weighed the baby 1  Asked about feeding 2  Asked about skin‐to‐skin practices 3  Checked baby’s temperature 4  Gave advice about caring for baby 5  Other (specify ) 6  Don’t know/Not sure 8 |
| **Acceptability of KMC** | | |
| **50.** | Overall, are you satisfied with your baby’s progress from practicing KMC? Explain why or why not | Yes (explain why below) 1  No (explain why not below) 2 |
| **51.** | Do you intend to continue practicing KMC? Why or why not? | Yes (explain why below) 1  No (explain why not below) 2 |
| **52.** | Would you recommend KMC to another woman who had a small/preterm baby? Why or why not? | Yes (explain why below) 1  No (explain why not below) 2 |

| **#** | **Item** | **Response** |
| --- | --- | --- |
| **Satisfaction with KMC wrap at home (both study arms)** | | |
| **53.** | Overall, how satisfied are you with the KMC wrap you received for use at home/in the community? Why or why not satisfied? | Very satisfied 1  Somewhat satisfied 2  Not very satisfied 3  Don’t know 8  Reason: |
| **54.** | Do you think the design of the wrap you received is acceptable for fathers or other male household members to use (gender neutral)? | Yes (explain why below) 1  No (explain why not below) 2 |
| **55.** | Have you used the KMC wrap you received for anything else (besides putting baby in skin‐to‐skin position)? | Yes (explain how used below) 1  No 2 |
| **56.** | Are you comfortable going outside your home with the baby in KMC position using the KMC wrap you received? Why or why not? | Yes (explain why below) 1  No (explain why not below) 2 |
| **57.** | Would you recommend the KMC wrap you received to other mothers for practicing KMC at home/in the community? Why or why not? | Yes (explain why below) 1  No (explain why not below) 2 |
| **58.** | Are you willing to return the KMC wrap you received so that it can be washed and recycled for other mothers to use it? If no, not? | Yes 1  No (explain why not below) 2 |
| **59.** | For this study, you were given a new wrap. If a new wrap had not been available, would you be willing to use a recycled wrap? If no, why not? | Yes 1  No (explain why not below) 2 |

| **#** | **Item** | **Response** |
| --- | --- | --- |
| **60.** | For this study, the wrap was given to you for free. If it had not been available for free, would you have been willing to buy it? | Yes (explain why below) 1  No (explain why not below) 2 >>#62 |
| **61.** | How much would you be willing to pay for the wrap? | 🗌🗌🗌🗌 kwacha  Not sure 99 |
| **62.** | Do you think other mothers with LBW babies will purchase the wrap to do KMC for their babies? | Yes… 1  No… 2  Not sure. 3 |
| **63.** | Would you have been willing to pay a small deposit for the wrap (200 kwacha or less) that would be given back to you when you returned the wrap? | Yes (explain why below) 1  No (explain why not below) 2 |
| **Question for mothers in intervention cohort only (LGH wrap)** | | |
| **64.** | Do you prefer the KMC wrapper over the traditional chitenje for practicing KMC at home/in the community? Why or why not? | Yes (explain why below) 1  No (explain why not below) 2  Don’t know/Not sure… 3 |
| *Thank participant for the continuing support from the beginning of the study and ask if she wants to share something important about the perception, usefulness and challenges or story/ verbatim of KMC practice and wrap that she might think has not been covered by the conversation.*  **………………………………………………………………………………………………………………………**  **……………………………………………………………………………………………………………………** | | |

**I have checked the form for missing variable and inconsistencies:**

| **65.** | Form checked by research coordinator |  | Date *(dd/mm/yyyy)* | / / |
| --- | --- | --- | --- | --- |
| **66.** | Data Entry 1 (Editor) |  | Date *(dd/mm/yyyy)* | / / |
| **67.** | Data Entry 2 |  | Date *(dd/mm/yyyy)* | / / |
