# Supplementary material for: Improving Skin-to-Skin Practice for babies in Kangaroo Mother Care in Malawi through the use of a customized baby wrap: A randomized control trial
Source: PLoS One. 2020 Mar 19;15(3):e0229720. doi: 10.1371/journal.pone.0229720 (PMC7082027; doi:10.1371/journal.pone.0229720)
Supplement: S3 Appendix — (DOCX) [file pone.0229720.s003.docx]

### S3 Appendix: Background characteristics of enrolled mothers

Table 1 details the background characteristics of enrolled mothers, and it shows that there were no significant differences between study arms at enrollment. The mean age of mothers was 25.1 years; nearly one quarter of mothers were less than 20 years of age. Education levels were low, with less than 60% of mothers having completed primary school. The majority of mothers were married, and just over half were unemployed. Monthly family income levels were low, with more than 80% of mothers reporting less than 30,000 kwacha (about US$41.4) per month and 38% reporting less than 10,000 kwacha (about US$13.8) per month.

Table 1. Background characteristics of mothers enrolled in the study

| Background characteristics of mothers enrolled in the study | CarePlus  (*n*=152) | *Chitenje*  (*n*=149) | Total  (*N*=301) | | *p*-value |
| --- | --- | --- | --- | --- | --- |
| Study site |  |  |  | |  |
| QECH | 38.2 | 40.3 | 39.2 | | NA |
| Thyolo | 27.6 | 26.9 | 27.2 | |  |
| Machinga | 34.2 | 32.9 | 33.6 | |  |
| Age |  |  |  | |  |
| Less than 20 years | 21.7 | 27.5 | 24.6 | | 0.47 |
| 20-29 years | 42.8 | 42.3 | 42.5 |  | |
| 30+ years | 34.9 | 30.2 | 32.6 | |  |
| Education level |  |  |  | |  |
| Less than primary | 55.3 | 60.4 | 57.8 | | 0.45 |
| Completed primary | 27.0 | 20.8 | 23.9 | |  |
| Completed some secondary or higher | 17.8 | 18.8 | 18.2 | |  |
| Marital status |  |  |  | |  |
| Married | 75.7 | 80.5 | 78.1 | | 0.31 |
| Not married | 24.3 | 19.5 | 21.9 | |  |
| Employment status |  |  |  | |  |
| None | 48.3 | 57.3 | 52.7 | | 0.32 |
| Skilled/self-employed | 4.2 | 3.6 | 3.9 | |  |
| Salaried | 9.8 | 4.5 | 7.1 | |  |
| Agriculture/farmer | 15.4 | 17.4 | 16.4 | |  |
| Business | 21.7 | 15.9 | 18.9 | |  |
| Other | 0.7 | 1.5 | 1.1 | |  |
| Monthly family income |  |  |  | |  |
| <10,000 kwacha | 37.5 | 39.6 | 38.5 | | 0.97 |
| 10-30,000 kwacha | 44.7 | 42.3 | 43.5 | |  |
| >30,000+ kwacha | 17.8 | 18.1 | 17.9 | |  |

Table 2 summarizes the health characteristics of mothers and babies enrolled in the study, as documented in maternity records. No significant differences were seen between study arms. For about two thirds of mothers, this was not their first child. The majority gave birth within a facility, with about 69% delivering in the study facility and 17% at another facility. An estimated 24% of mothers were known to be HIV+, 75% were known HIV-, and 1% had unknown status. Maternal complications were quite common, with close to one quarter of enrolled mothers documented in maternity ward records as having experienced at least one maternal complication. Preeclampsia was the most frequently documented complication (10.0%; 30/301), followed by hemorrhage (5.0%; 14/301), prolonged labor (1.3%; 4/301), and other (9.0%; 27/301).

Table 2. Health characteristics of study mothers and babies at enrollment

| Health characteristics of study mothers and babies at enrollment | CarePlus (*n*=152) | *Chitenje*  (*n*=149) | Total  (*N*=301) | *p*-value |
| --- | --- | --- | --- | --- |
| Mothers: |  |  |  |  |
| Parity |  |  |  |  |
| First baby | 30.9 | 31.5 | 31.2 | 0.91 |
| Second+ baby | 69.1 | 68.5 | 68.8 |  |
| Place of delivery |  |  |  |  |
| Study facility | 67.7 | 71.4 | 69.4 | 0.50 |
| Other health facility | 15.8 | 18.1 | 16.9 |  |
| Outside health facility | 15.1 | 9.4 | 12.3 |  |
| Maternal complications |  |  |  |  |
| None | 57.9 | 55.7 | 56.8 | 0.42 |
| One or more | 25.7 | 22.2 | 23.9 |  |
| Unknown | 16.5 | 22.2 | 19.3 |  |
| Maternal HIV status |  |  |  |  |
| HIV+ | 22.7 | 24.5 | 23.6 | 0.77 |
| HIV- | 76.7 | 74.2 | 75.4 |  |
| Unknown | 0.7 | 1.4 | 1.0 |  |
| Babies: |  |  |  |  |
| Sex |  |  |  |  |
| Male | 43.3 | 42.2 | 42.8 | 0.84 |
| Female | 56.7 | 57.8 | 57.2 |  |
| Mode of delivery |  |  |  |  |
| Vaginal | 84.0 | 80.3 | 82.2 | 0.46 |
| C-section | 16.0 | 19.1 | 17.5 |  |
| Vacuum extraction | 0.0 | 0.7 | 0.3 |  |
| Gestational age (completed weeks) |  |  |  |  |
| <32 weeks | 29.0 | 20.8 | 24.9 | 0.17 |
| 32-36 weeks | 63.2 | 67.1 | 65.1 |  |
| 37+ weeks | 7.9 | 12.1 | 10.0 |  |
| Multiple births |  |  |  |  |
| Singleton | 69.7 | 65.8 | 67.8 | 0.59 |
| Twins/Triplets | 29.0 | 31.5 | 30.2 |  |
| Birthweight (as measured in maternity ward) |  |  |  |  |
| <1,500g | 38.8 | 34.9 | 36.9 | 0.48 |
| 1,500-<2,000g | 61.2 | 65.1 | 63.1 |  |
| Other complications at birth (as recorded in maternity ward) |  |  |  |  |
| None | 86.8 | 85.2 | 86.1 | 0.69 |
| Asphyxia | 2.0 | 3.4 | 2.7 | 0.46 |
| Sepsis | 0 | 0 | 0 | -- |
| Other | 11.2 | 11.4 | 11.3 | 0.95 |

Most babies were delivered vaginally, while 17.5% were delivered through c-section. Only 10% of enrolled babies were estimated to be term (37+ weeks), with close to two thirds estimated to be between 32 and 36 weeks gestation. An estimated 63% of babies were born weighing between 1,500 and 2,000g, while about 37% were less than 1,500g at birth. Less than 15% of babies were documented to have experienced other complications at birth (in addition to preterm/low birth weight), with asphyxia being the most common.

The average age in days at admission to KMC was 1.5, and it was similar in both study arms (Table 3a). The average weight at initiation to KMC was 1,555g, and it was similar in both study arms. More than one third of babies (36.2%) were less than 1,500g at initiation, and about 20% were between 1,800 and 2,000g. Duration of stay in KMC was 9.3 days on average, with babies in the intervention arm staying slightly longer on average than control babies, although the difference was not statistically significant. Close to 20% of babies became sick while in facility-KMC, with injections and phototherapy being the most common treatments received.

Nearly all babies were discharged from facility alive, with 14 deaths recorded in facility-KMC (six in intervention and eight in control). About two thirds (67.8%) of babies met discharge criteria, while 23.6% left against medical advice and the status of 3.7% was unknown. Weight at discharge from facility was missing for 3.8% of cases, with slightly more *chitenje* cases missing data than women using the CarePlus Wrap. Among those with known weight at discharge (*n*=276; 96.2% of the 287 discharged alive), average weight was similar between groups, with the difference between average weight at initiation and discharge being 62g (63g in the CarePlus group and 60g in the *chitenje* group). Just over 20% of babies were discharged from the facility weighing less than 1,500g.

Table 3a. Summary of KMC experience among enrolled mothers and babies by study arm

|  | CarePlus (*n*=152) | *Chitenje*  (*n*=149) | Total  (*N*=301) | *p*-value |
| --- | --- | --- | --- | --- |
| Age in days at admission to KMC | 1.5 | 1.6 | 1.5 | 0.95 |
| Weight at KMC initiation (mean) | 1,547g | 1,563g | 1,555g | 0.58 |
| <1,500g | 40.1 | 32.2 | 36.2 | 0.26 |
| 1,500-1,799g | 39.5 | 48.3 | 43.9 |  |
| 1,800g-<2,000g | 20.4 | 19.5 | 19.9 |  |
| Status at discharge from KMC (%) |  |  |  |  |
| Met discharge criteria | 66.5 | 69.1 | 67.8 | 0.64 |
| Died | 4.0 | 5.4 | 4.7 |  |
| Referred to higher level | 0.0 | 0.7 | 0.3 |  |
| Left against medical advice | 26.3 | 20.8 | 23.6 |  |
| *Unknown* | 3.0 | 4.0 | 3.7 |  |
| Weight at KMC discharge for those discharged alive (mean) | 1,610g | 1,623g | 1,617g | 0.60 |
| <1,500g | 24.7 | 18.4 | 21.6 | 0.17 |
| 1,500-1,799g | 51.4 | 58.2 | 54.7 |  |
| ≥1,800g | 21.9 | 17.7 | 19.7 |  |
| *Missing* | *2.1* | *5.7* | *3.8* |  |
| Total number of days in KMC (mean) | 9.7 | 8.9 | 9.3 | 0.38 |
| *Missing* | *12 cases* | *17 cases* | *29 cases* |  |
| Child age in days at time of discharge | 11.1 | 9.7 | 10.4 | 0.13 |
| *Missing/unknown* | *21 cases* | *31 cases* | *52 cases* |  |
| Child became sick while in KMC | 22.2 | 17.4 | 19.9 | 0.35 |
| Treatment received while in KMC |  |  |  |  |
| Injections (antibiotics) | 13.2 | 9.4 | 11.3 | 0.30 |
| Phototherapy | 4.0 | 4.0 | 4.0 | 0.97 |
| Other | 1.3 | 0.7 | 1.0 | 0.57 |

Analysis by study site revealed significant differences across study sites (Table 3b). Babies at QECH tended to be smaller at time of initiation, but weight gains were greater, and a larger proportion met discharge criteria than at the other two sites. Babies at Thyolo lost weight on average between admission and facility discharge, and only about half of those admitted met discharge criteria; most concerning, 9.8% died while in facility-KMC, more than one quarter left against medical advice, and status at discharge was unknown for another 9.8%. Weight at discharge was missing for 4.4% of the babies discharged alive from QECH, 1.0% of babies at Machinga, and 6.8% at Thyolo.

Table 3b. Summary of KMC experience among enrolled mothers and babies by study site

| Summary of KMC experience among enrolled mothers and babies | QECH  (*n*=118) | Thyolo  (*n*=82) | Machinga  (*n*=101) | *p*-value |
| --- | --- | --- | --- | --- |
| Age in days at admission to KMC | 2.2 | 1.2 | 1.1 | 0.01 |
| Weight at KMC initiation (mean) | 1,488g | 1,602g | 1,593g | 0.00 |
| <1,500g | 44.1 | 31.7 | 30.7 | 0.01 |
| 1,500-1,799g | 46.6 | 40.2 | 43.6 |  |
| 1,800g-<2,000g | 9.3 | 28.1 | 25.7 |  |
| Status at discharge from KMC |  |  |  |  |
| Met discharge criteria | 74.6 | 51.2 | 73.3 | 0.00 |
| Died | 2.5 | 9.8 | 3.0 |  |
| Referred to higher level | 0.0 | 1.2 | 0.0 |  |
| Left against medical advice | 21.2 | 28.1 | 22.8 |  |
| Unknown | 1.8 | 9.8 | 1.0 |  |
| Weight at KMC discharge among those discharged alive (mean) | 1,637g | 1,577g | 1,622g | 0.15 |
| <1,500g | 15.7 | 32.4 | 20.4 | 0.02 |
| 1,500-1,799g | 63.5 | 43.2 | 53.1 |  |
| ≥1,800g | 16.5 | 17.6 | 25.5 |  |
| *Missing* | *4.4* | *6.8* | *1.0* |  |
| Total number of days in KMC (mean) | 8.9 | 7.4 | 10.9 | 0.01 |
| *Missing* | *8 cases* | *19 cases* | *2 cases* |  |
| Child age in days at time of discharge | 10.9 | 7.7 | 11.8 | 0.00 |
| *Missing/unknown* | *20 cases* | *21 cases* | *11 cases* |  |
| Child became sick while in KMC | 22.5 | 11.9 | 22.6 | 0.20 |
| Treatment received while in KMC |  |  |  |  |
| Injections (antibiotics) | 5.8 | 2.6 | 9.6 | 0.01 |
| Phototherapy | 5.8 | 0.9 | 0.5 | 0.00 |
| Other | 0.7 | 0.9 | 0.0 | 0.44 |
